# Exploring membranous NECTIN‐4 expression patterns and enfortumab vedotin response in prostate cancer

**DOI:** 10.1111/jcmm.18572

**Published:** 2024-07-28

**Authors:** Richard Weiten, Marit Bernhardt, Max Niemann, Glen Kristiansen, Viktor Grünwald, Manuel Ritter, Michael Hölzel, Markus Eckstein, Abdullah Alajati, Niklas Klümper, Philipp Krausewitz

**Affiliations:** ^1^ Department of Urology and Paediatric Urology University Hospital Bonn Bonn Germany; ^2^ Department of Urology Uro‐Oncology, Robot‐Assisted and Specialized Urologic Surgery University Hospital Cologne Koln Germany; ^3^ Institute of Pathology University Hospital Bonn Bonn Germany; ^4^ Clinic for Internal Medicine (Tumor Research) and Clinic for Urology, Interdisciplinary Genitourinary Oncology at the West‐German Cancer Center Essen University Hospital Essen Germany; ^5^ Institute of Experimental Oncology University Hospital Bonn Bonn Germany; ^6^ Institute of Pathology, University Hospital Erlangen Friedrich‐Alexander‐Universität Erlangen‐Nürnberg Erlangen Germany

**Keywords:** antibody‐drug conjugates, enfortumab vedotin, mCRPC, NECTIN‐4, prostate cancer

## Abstract

Antibody‐drug conjugates (ADCs) represent a novel type of targeted cancer therapy combining the specificity of monoclonal antibodies with the cytotoxicity of conventional chemotherapy. Recently, ADCs have demonstrated practice‐changing efficacy across diverse solid cancers. The anti‐NECTIN‐4 ADC enfortumab vedotin (EV) has just been approved for patients with urothelial cancer and is currently under investigation for patients with castration‐resistant prostate cancer (CRPC e.g. Phase II ENCORE trial). Our objective was to evaluate the efficacy of EV in established prostate cancer (PCa) cell lines and to examine the membranous NECTIN‐4 expression in primary tumours (PRIM) and distant metastases (MET). NECTIN‐4 was heterogeneously expressed in the panel of PCa cell lines. EV led to growth inhibition in NECTIN‐4 expressing PCa cells (22Rv1 and LNCaP), whereas the NECTIN‐4‐negative PC‐3 cells were significantly less responsive to EV, emphasizing the dependence of EV response on its target expression. Immunohistochemical staining revealed moderate membranous NECTIN‐4 expression only in a small subgroup of CRPC patients with lung and peritoneal MET [*n* = 3/22 with H‐score ≥100, median H‐score 140 (IQR 130–150)], while 100% of PRIM (*n* = 48/48) and 86.4% of common MET sites (*n* = 19/22), including lymph node, bone and liver MET, were NECTIN‐4 negative. In summary, EV may be effective in NECTIN‐4‐positive PCa. However, our findings demonstrate that the tumoural NECTIN‐4 expression is predominantly low in metastatic PCa, which suggests that EV may only be effective in a biomarker‐stratified subgroup.

## 1 INTRODUCTION

Patients with metastatic prostate cancer (mPCa) face a clinical challenge, characterized by limited therapeutic alternatives and a worsened prognosis.^1^ Overall survival (OS) is intricately tied to the locations of metastatic lesions, with lung and liver metastases exhibiting heightened lethality compared to bone and non‐visceral involvement.^2^

Despite promising novel anti‐androgenic therapies,^3^ poly (adenosine diphosphate‐ribose) polymerase (PARP) inhibitors such as olaparib^4^ and radioligand therapies like ^177^Lu‐PSMA,^5^ further improvements in the treatment of metastatic castration‐resistant prostate cancer (mCRPC) are warranted. In this context, antibody‐drug conjugates (ADCs) have emerged as a promising new class of targeted therapeutic agents, exhibiting remarkable efficacy in the treatment of metastatic solid tumours. The antibodies in ADCs are designed to bind specifically to proteins that are overexpressed on the surface of cancer cells, while the conjugated toxic payload subsequently kills those cells selectively.^6^, ^7^, ^8^ This targeted tumour delivery system is engineered to minimize off‐target toxicities in patients by restricting the exposure of normal tissues to the active cytotoxic component, for example, Monomethyl auristatin E (MMAE).^6^, ^7^, ^8^

Previous studies have demonstrated that NECTIN‐4 is overexpressed in various human solid tumours including breast, lung, pancreatic, oesophageal, ovarian and in particular urothelial cancer.^9^, ^10^ In addition, overexpression of NECTIN‐4 is associated with an increased risk of disease progression and unfavourable clinical outcomes.^9^, ^10^, ^11^ Therefore, NECTIN‐4 emerges as a promising diagnostic and therapeutic target in different types of cancers.^11^

The efficacy of enfortumab vedotin (EV), an NECTIN‐4‐targeted ADC, with proven efficacy for the treatment of patients with metastatic urothelial carcinoma (mUC), is currently being evaluated in the ENCORE phase II trial (NCT04754191) for CRPC.^12^ However, it has already been demonstrated in other tumour entities within the urological domain that the success of NECTIN‐4‐targeted therapies is dependent upon the expression profile of the target cell, which proves to be both heterogeneous and dynamic.^12^, ^13^ Our primary objective was to assess the efficacy of EV in a panel of PCa cell lines to establish a preclinical rationale for determining whether NECTIN‐4 overexpression is a constant mechanism within the context of castration resistance. Our secondary objective was to investigate the membranous NECTIN‐4 expression across various stages of PCa, guided by insights from mUC, emphasizing the importance of considering diverse expression patterns before implementing EV in clinical contexts.^13^

## 2 MATERIALS AND METHODS

### 2.1 Cell lines and culture conditions

Human PCa cell lines 22Rv1 (derived from a xenograft, CWR22R), PC‐3 (representing a bone metastasis), DU145 (representing a brain metastasis), LNCaP and C4‐2B (representing a lymph node metastasis) and BPH‐1 (representing a benign prostatic hyperplasia) provided by American Type Culture Collection (ATCC, Manassas, Virginia) were cultured in RPMI 1640 (#31870025, Thermo Fisher Scientific, Darmstadt, Germany) or DMEM medium (#11960044, Thermo Fisher Scientific, Darmstadt, Germany) supplemented with 10% heat‐inactivated foetal calf serum, 0.8% streptomycin–penicillin antibiotics (10.000 units/mL Penicillin and 10.000 μg/mL Streptomycin; #15140‐122, Thermo Fisher Scientific, Darmstadt, Germany) and 1% L‐glutamine (200 mM; #25030‐024, Thermo Fisher Scientific, Darmstadt, Germany). The cell cultures were incubated at 37°C in a humid environment with 5% carbon dioxide.

### 2.2 Western blot

Human PCa cell lines (22Rv1, C4‐2B, PC‐3, DU145, LNCaP) and a benign prostatic hyperplasia cell line (BPH‐1) were plated in six‐well plates reached 80%–90% confluency. Cells were then collected and lysed with RIPA lysis buffer containing protease inhibitors. Protein concentration was determined using the BCA protein assay (#23225, Pierce BCA Protein Assay Kit, Thermo Fisher Scientific, Darmstadt, Germany), then samples were added with 4x SDS sample loading buffer [Tris–HCl (0.2 mol/L), DTT (0.4 mol/L), SDS (277 mmol/L), 8.0% (w/v) Bromophenol blue (6 mmol/L), Glycerol (4.3 mol/L)] and denatured at 95°C for 5 min. The denatured samples were separated with a 4% SDS gel and transferred to a 0.45 μm nitrocellulose membrane (#GE10600002, Amersham Protran Premium Western Blotting Membrane, Merck, Darmstadt, Germany). The membrane was blocked with 5% non‐fat milk in TBST (50 nM Tris, 150 nM NaCl, 0.05% Tween 20, pH 7.5) for 60 min and then incubated overnight at 4°C with primary antibodies against NECTIN‐4 (#AF2659, R&D Systems, Wiesbaden‐Nordenstadt, Germany, dilution 1:500) and β‐Actin (#A2228, Sigma dilution, Taufkirchen, Germany, dilution 1:5000). HRP‐linked secondary antibody against mouse (#170–6516, Bio‐Rad, Feldkirchen, Germany) were applied for 1 h in TBST. The fluorescence signal was detected using the ChemiDoc MP Imaging System (Bio‐Rad, Feldkirchen, Germany).

### 2.3 Flow cytometry

The immunostaining procedure was performed according to standard protocols. Single‐cell suspensions of DU145, PC‐3, LNCaP, 22Rv1 and BPH‐1 were stained with the anti‐human NECTIN‐4 antibody (#130‐116‐028, Miltenyi Biotec, Bergisch Gladbach, Germany, dilution 1:100) and Zombie NIR (#423106, BioLegend, San Diego, CA, USA, dilution 1:400). Data were collected using a BD FACSCanto II flow cytometer (BD Biosciences, Heidelberg, Germany) and analysed using FlowJo software (FlowJo v10.8 BD, https://www.flowjo.com). A total of 1 × 10^4^ cells were measured for each sample.

### 2.4 Measurement of cell viability

To measure the viability of cells after treatment with the NECTIN‐4‐targeted ADC enfortumab vedotin (EV), we used a crystal violet assay (0.05% crystal violet stain, 0.1% acetic acid). In this assay, cells (1 × 10^4^) were exposed to different concentrations of EV (0–20 μg/mL) for 48 h, fixing with 37% formaldehyde and stained with crystal violet based on previous publications.^14^ The absorbance of the stained cells was measured with an ultraviolet–visible spectrometer [570 nm, Safire Reader (Tecan), Männedorf, Switzerland] and the relative viability of the cells was calculated based on the absorbance values.

### 2.5 Patient cohort

We investigated a retrospective well‐characterized cohort with primary tumours (PRIM, *n* = 48) and metastatic disease (MET, *n* = 22; including lymph node, bone and visceral metastasis) treated at the Department of Urology at the University Hospital of Bonn and the University Hospital of Cologne (*n* = 70). Clinicopathologic data of the cohort are summarized in Tables S1 and S2. The histopathological diagnosis was based on the 8th TNM classification^15^ for malignant tumours and the 5th WHO classification for urogenital tumours.^16^

The study received approval from the ethical review boards of the Medical Faculty of the University of Bonn (approval numbers: 372/21) and the Medical Faculty of the University of Cologne (approval number: 23–1178). It was conducted in accordance with the Declaration of Helsinki, and all patients provided written informed consent.

### 2.6 Immunohistochemistry

NECTIN‐4 protein was detected by immunohistochemical (IHC) staining on a VENTANA BenchMark ULTRA autostainer (Ventana Medical System) according to an accredited staining protocol in a routine IHC laboratory. An anti‐NECTIN‐4 monoclonal antibody (#ab251110, Abcam, dilution of 1:800, incubated for 32 min at 37°C) was used as primary antibody. Antigen recovery was performed using Cc1 buffer from Ventana for 64 min at 91°C. Two independent pathologists (M. Bernhardt and Yuri Tolkach) evaluated H‐score for membranous NECTIN‐4 staining. Samples were classified as negative (H‐score 0–14), weak (H‐score 15–99), moderate (H‐score 100–199) and strong (H‐score 200–300), as previously described.^11^, ^13^

### 2.7 Statistical analysis

Statistical analysis was conducted using SPSS (Version 28.0.1.1) and GraphPad Prism (Version 9.4.0). The non‐parametric Mann–Whitney test and parametric *t*‐test were employed for comparing two groups, while the non‐parametric Kruskal–Wallis test was applied to compare multiple groups. All *p*‐values were calculated two‐sided and a *p* < 0.05 was considered statistically significant.

## 3 RESULTS

### 3.1 On‐target efficacy of EV in PCa cell lines

**FIGURE 1:**
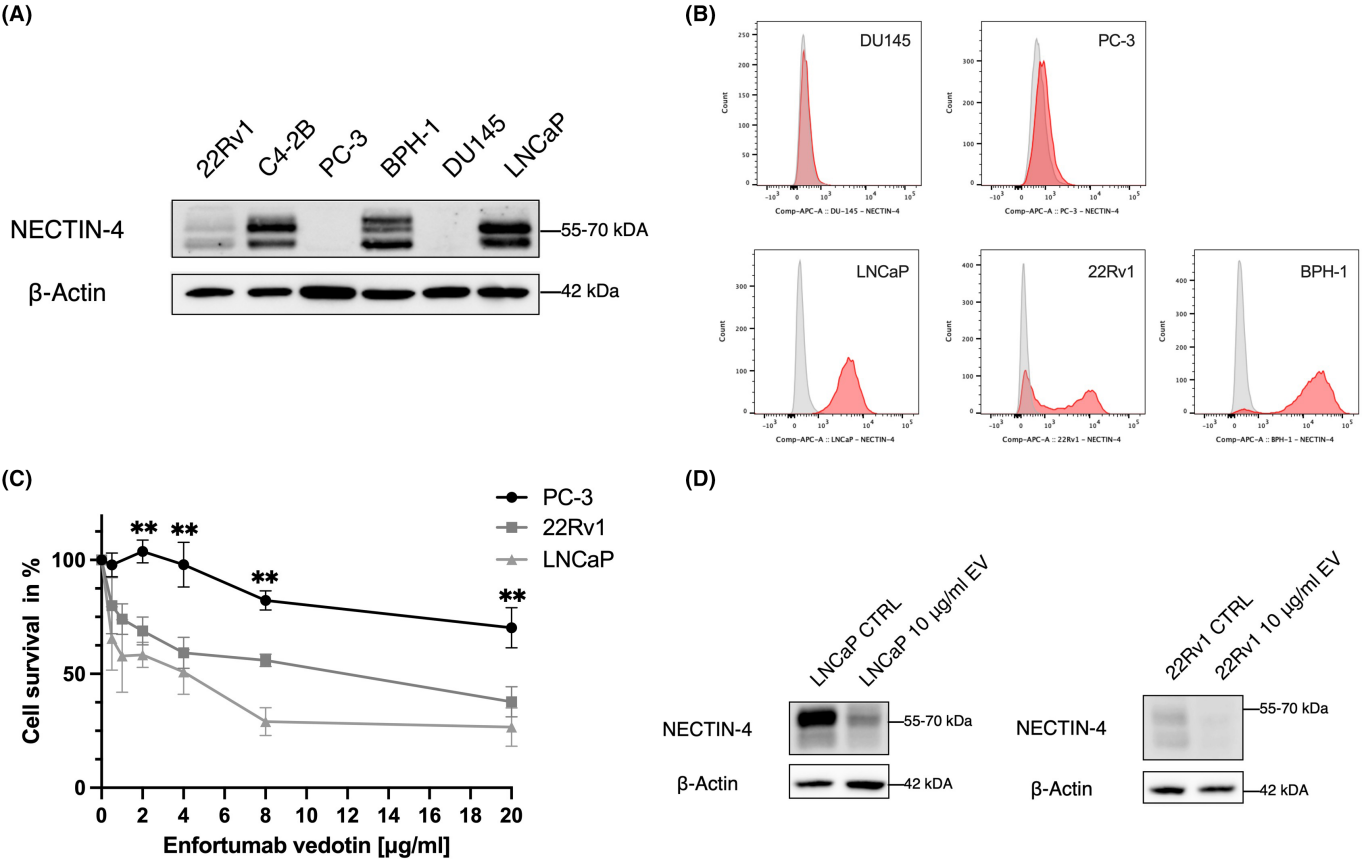
On‐target efficacy of enfortumab vedotin in prostate cancer cell lines. (A) NECTIN‐4 protein expression levels in a panel of PCa cell lines (22Rv1, C4‐2B, PC‐3, DU145 and LNCaP) using Western blot, with BPH‐1 (non‐malignant prostate cell line) 22Rv1, C4‐2B and LNCaP cells displaying a weak to strong NECTIN‐4 expression. Detection of b‐actin served as loading control. (B) Normalized histogram illustrating membranous NECTIN‐4 expression detected by flow cytometry in DU145, PC‐3, LNCaP, 22Rv1 and BPH‐1 (plus unstained control as reference). (C) EV led to significant growth inhibition in the NECTIN‐4‐expressing 22Rv1 and LNCaP, while NECTIN‐4‐negative PC‐3 cells were found to be resistant to EV treatment (*t*‐test, *p* < 0.01). **(**D) EV led to a reduced NECTIN‐4 expression in the NECTIN‐4 expressing PCa cell lines LNCaP and 22Rv1, shown by western blot. PCa, prostate cancer; EV, enfortumab vedotin. **p < 0.01.

We determined NECTIN‐4 expression levels through Western blot and flow cytometry in a panel of PCa cell lines (22Rv1, C4‐2B, PC‐3, DU145 and LNCaP) and in a benign prostatic hyperplasia cell line (BPH‐1). The human PCa cell lines C4‐2B and LNCaP and the non‐malignant PCa cell line BPH‐1 exhibited strong NECTIN‐4 protein expression, while 22Rv1 demonstrated only weak expression of NECTIN‐4. In contrast, both PC‐3 and DU145 cell lines were NECTIN‐4 negative (Figure 1A,B). Next, we investigated the efficacy of EV on the in vitro cell growth of PCa cells by exposing LNCaP, 22Rv1 and PC‐3 cells to different concentrations of EV (0–20 μg/mL). As shown in Figure 1C, EV induced dose‐dependent inhibition of cell growth of NECTIN‐4 expressing cancer cells (LNCaP and 22Rv1), while NECTIN‐4‐negative PC‐3 cells were resistant to EV treatment (unpaired *t*‐test, *p* < 0.01). In this context, the inhibitory effect of EV significantly correlated with NECTIN‐4 expression and EV doses (PC‐3, *R* ^2^ = 0.75; 22Rv1, *R* ^2^ = 0.67; and LNCaP, *R* ^2^ = 0.52). Western blot analysis confirmed a substantial decrease in NECTIN‐4 protein expression in EV‐treated NECTIN‐4‐positive cells (LNCaP and 22Rv1 10 μg/mL EV) compared to non‐treated cells (LNCaP and 22Rv1 CTRL) (Figure 1D).

### 3.2 Heterogenous membranous NECTIN‐4 expression pattern

**FIGURE 2:**
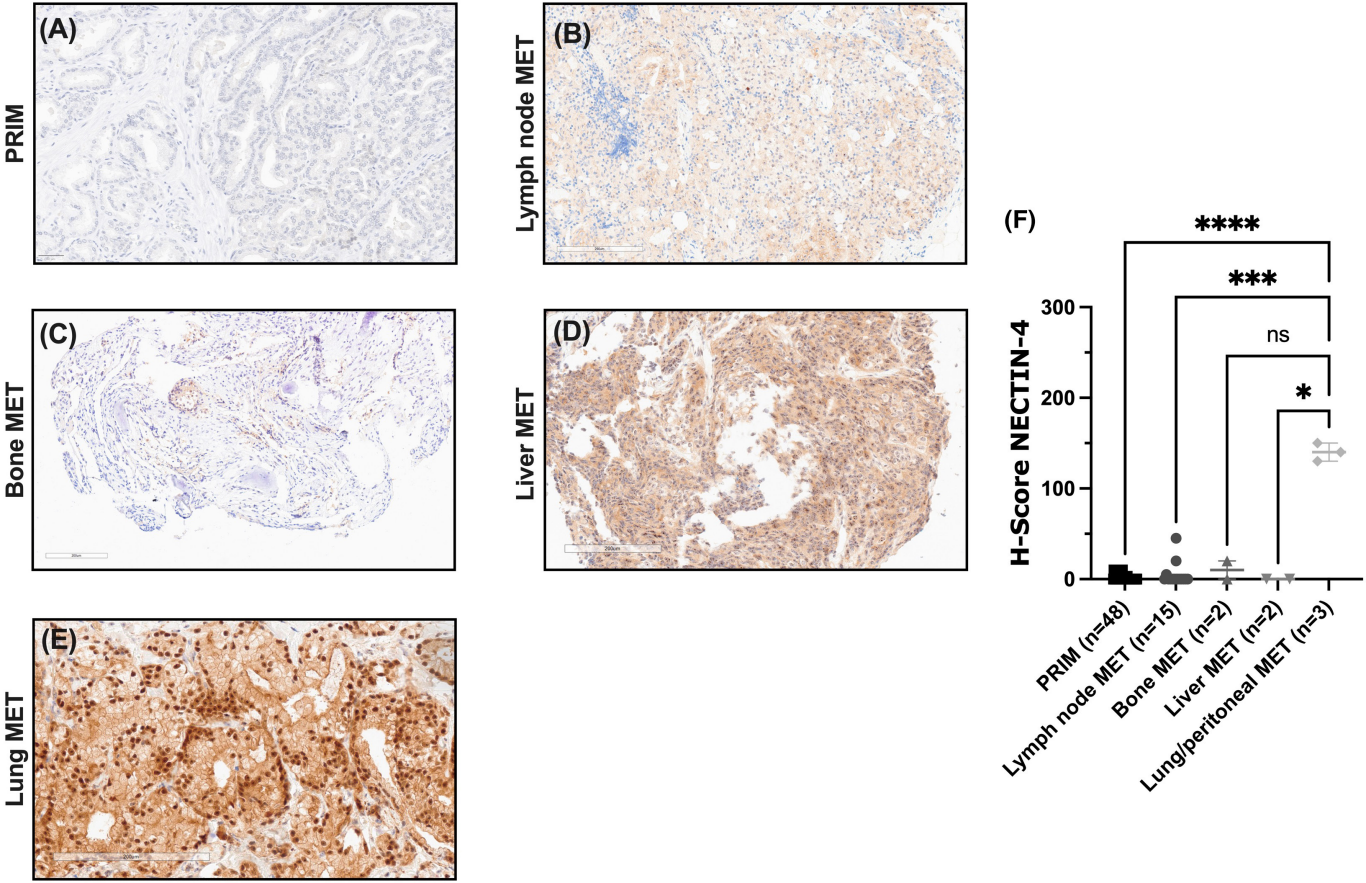
Membranous NECTIN‐4 protein expression patterns assessed by immunohistochemistry. (A–E) Representative immunohistochemical images of PRIM and different MET sites, with no or weak membranous NECTIN‐4 expression in PRIM (A, H‐score 0), lymph node (B, H‐score 5), bone (C, H‐score 10) and liver MET (D, H‐score 0), while NECTIN‐4 was moderately expressed in lung MET (E, H‐score 140) (200 × magnification). (F) Moderate membranous NECTIN‐4 expression in lung and peritoneal MET (*n* = 3/3) [median H‐score = 140 (IQR 130–150)], while 100% of PRIM and 86.4% of MET, including lymph node, bone and liver MET, were NECTIN‐4‐negative (Kruskal–Wallis test, *p < 0.05, ***, *p* < 0.001, ****p < 0.0001). IQR, interquartile range; MET, metastases.

Immunohistochemical analysis, followed by an evaluation of the H‐score, showed heterogeneous NECTIN‐4 expression pattern across primary tumours and distant metastases, including lymph node, bone and visceral metastases. Representative immunohistochemical images are depicted in Figure 2A–E. Notably, specific immunoreactivity for NECTIN‐4 was detected predominately on cell membrane and cytoplasm of tumour cells, while the surrounding normal tissue was NECTIN‐4 negative. However, only specific membranous expression was assessed as the biological prerequisite for EV binding.

Despite the relatively small number of only three cases of lung and peritoneal MET, all of them revealed moderate membranous NECTIN‐4 expression, defined as an H‐score ≥100, as previously described,^11^, ^13^ with a median H‐score of 140 [interquartile range (IQR) 130–150] (Kruskal–Wallis test, *p* < 0.0001) (Figure 2E). In contrast, 100% of PRIM (*n* = 48/48) were classified as NECTIN‐4 negative, and thus, no association was found with established clinicopathological parameters (Figure 2A). Furthermore, NECTIN‐4 exhibited negligible or minimal expression in prevalent metastatic sites such as lymph nodes and bone metastases, characterized by a median H‐score of 0 (IQR, 0) and 10 (IQR, 0–20), respectively. Similarly, in liver metastases, the expression of NECTIN‐4 was minimal, reflected by a median H‐score of 0 (IQR, 0) (Figure 2B–D). Notably, there were no differences in NECTIN‐4 expression between hormone‐sensitive and castration‐resistant PCa.

## 4 DISCUSSION

Due to the recent successes of EV in mUC (EV‐301 and EV‐302 trial),^12^, ^17^ and the overexpression of NECTIN‐4 in various human malignancies,^9^, ^10^ our objective was to scrutinize the expression of NECTIN‐4 in metastatic PCa. Our objectives were dual‐fold: first, to establish a preclinical rationale for utilizing EV in metastatic PCa, and second, to explore the NECTIN‐4 expression across different stages of the disease in order to identify subgroups who most likely benefit from EV treatment.

Through our investigations involving established PCa cell lines and human PCa samples, we were able to formulate two key statements: First, NECTIN‐4 is heterogeneously expressed in vitro and in vivo in PCa. Second, EV inhibits growth in NECTIN‐4 expressing cell lines, providing a preclinical rationale for its application in patients with metastatic PCa. In this context, NECTIN‐4 expression does not appear to represent a general mechanism in the context of castration resistance, as most metastatic sites exhibited weak or no NECTIN‐4 expression. Furthermore, our results align with previous research indicating the absence of NECTIN‐4 in PRIM and its expression in benign prostatic hyperplasia.^18^ We also demonstrate that NECTIN‐4 expression serves as a crucial determinant in predicting the therapeutic efficacy of EV in PCa patients. Therefore, as observed in mUC,^14^ diverse expression patterns must be considered in PCa patients too. In accordance with our findings revealing limited NECTIN‐4 expression in prostate cancer metastases, a thorough evaluation of NECTIN‐4 expression as a predictive marker is granted in EV‐treated patients with mCRPC. This is especially important, as mCRPC prognosis is strongly influenced by the pattern of metastasis,^2^ and EV response is highly dependent on the membranous NECTIN‐4 expression, which represent the biological prerequisite as demonstrated in mUC.^13^ Building upon these insights and supported by our promising in vitro results, which indicated a potential application of EV in PCa patients with NECTIN‐4 overexpression, we systematically investigated diverse metastatic sites, encompassing bone, lymph node and visceral metastases. Yet, NECTIN‐4 positivity was solely detected in infrequently occurring lung and peritoneal metastases. As a result, a general applicability or a specific pattern of EV efficacy in mPCa remains elusive for now, with its benefits confined limited to individual cases. However, these assumptions must be made with caution, as they are based on a limited case sample that prohibits generalization.

On the contrary, our results highlight the potential benefit for patients with NECTIN‐4 expression in their tumour tissue. This emphasizes the importance of assessing NECTIN‐4 status in men with metastatic PCa before considering potential EV treatment. This recommendation aligns with Klümper et al.'s findings, which indicate that the absence or weak membranous NECTIN‐4 expression in mUC predicts resistance to EV and is linked to unfavourable outcomes.^13^, ^19^

NECTIN‐4 serum levels are elevated in patients with non‐small cell lung, breast and ovarian cancers.^20^, ^21^, ^22^ This suggests that NECTIN‐4 status in serum could be a potential predictive biomarker for predicting therapeutic response to EV. However, further investigations are required to establish the correlation between NECTIN‐4 expression in metastatic sites and NECTIN‐4 expression on CTCs. Herein, molecular imaging holds great potential for providing valuable information on the ADC target expression status, as has been shown for NECTIN‐4 in urothelial cancer,^23^, ^24^ enabling for more targeted and personalized treatment decisions in terms of ADC precision oncology. Moreover, clinical trials, like the phase II ENCORE trial (NCT04754191) evaluating EV in patients with mCRPC, are needed to thoroughly assess the efficacy and safety of EV.

Besides the complexity of translating in vitro findings into clinical practice, retrospective data collection may lead to potential bias. In addition, the size of metastatic samples used in this study was small, and the primary and metastatic samples were not from the same patient. As a result, there may be differences in prior treatment history, tumour aggressiveness and patient demographics. Hence, validation of our results in larger cohorts is crucial for establishing the clinical relevance of NECTIN‐4 as a predictive biomarker and understanding the broader implications of a potential EV treatment in mPCa patients. Moreover, we cannot make definitive mechanistic assertions. Nevertheless, even within our small sample size, it becomes evident that the application of NECTIN‐4 targeted ADC in patients with metastatic PCa without testing for its target expression at metastatic sites is likely to yield limited success. Therefore, it will be intriguing to observe the first results of the ENCORE trial.

Another important limitation of this study is the heterogeneity of our patient cohort, especially the limited representation of bone metastases, which are common in a significant proportion of metastatic patients. Therefore, the generalizability of our findings is limited, and further studies with more diverse patient populations are needed to comprehensively validate our results.

## 5 CONCLUSION

Our results suggest that EV may be particularly effective in treating mCRPC patients with moderate to strong membranous NECTIN‐4 expression. However, the findings also demonstrate that tumoural NECTIN‐4 expression is heterogeneous in mCRPC patients, advocating for pretherapeutic biomarker assessment before considering in vivo application to maximize the efficacy of this promising new drug.

## AUTHOR CONTRIBUTIONS

**Richard Weiten:** Conceptualization (lead); data curation (equal); formal analysis (equal); investigation (equal); methodology (equal); project administration (equal); resources (equal); software (equal); supervision (equal); writing – original draft (lead). **Marit Bernhardt:** Formal analysis (equal); methodology (supporting); writing – review and editing (equal). **Max Niemann:** Data curation (equal); investigation (equal); writing – review and editing (equal). **Glen Kristiansen:** Resources (supporting); writing – review and editing (equal). **Viktor Grünwald:** Writing – review and editing (equal). **Manuel Ritter:** Resources (supporting); writing – review and editing (equal). **Michael Hölzel:** Writing – review and editing (equal). **Markus Eckstein:** Formal analysis (supporting); writing – review and editing (equal). **Abdullah Alajati:** Resources (supporting); writing – review and editing (equal). **Niklas Klümper:** Project administration (supporting); supervision (supporting); writing – review and editing (equal). **Philipp Krausewitz:** Conceptualization (equal); project administration (equal); supervision (lead); writing – original draft (equal).

## FUNDING INFORMATION

This research received no specific grant from any funding agency in the public, commercial or not‐for‐profit sectors.

## CONFLICT OF INTEREST STATEMENT

VG: Research funding from AstraZeneca, Novartis, BMS, MSD, Ipsen, Pfizer; honoraria and consultation fees from AstraZeneca, BMS, Novartis, Amgen, Astellas, Apogepha, Ipsen, EISAI, MSD, MerckSerono, Roche, EUSAPharm, Janssen, ONO Pharmaceutical, cureteq, Debiopharm, PCI Biotech, Oncorena, Novartis/AAA, Gilead; stocks shareholder from AstraZeneca, BMS, SeaGen, MSD, GenMab; travel expenses AstraZeneca, BMS, MerckSerono; Janssen. MR: Speaker's honoraria from medac; advisory role for Siemens Healthineers; research funding from Procept BioRobotics. MH: Research funding by TME Pharma (Noxxon), honoraria from BMS, Novartis. ME: Personal fees, travel costs and speaker's honoraria from MSD, AstraZeneca, Janssen‐Cilag, Cepheid, Roche, Astellas, Diaceutics; research funding from AstraZeneca, Janssen‐Cilag, STRATIFYER, Cepheid, Roche, Gilead; advisory roles for Diaceutics, MSD, AstraZeneca, Janssen‐Cilag, GenomicHealth. NK: Personal fees, travel costs and speaker's honoraria from Astellas, Novartis, Ipsen, Photocure, MSD, Eisai. PK: Personal fees, travel costs and speaker's honoraria from Bayer, Janssen‐Cilag, Medac, Novartis. All others authors declare no conflict of interest.

## PATIENT CONSENT STATEMENT

All patients provided written informed consent.

## Supporting information


Table S1.

## Data Availability

The data that support the findings of this study are available from the corresponding author upon reasonable request.
