# Supplementary material for: Exploring membranous NECTIN‐4 expression patterns and enfortumab vedotin response in prostate cancer
Source: J Cell Mol Med. 2024 Jul 28;28(14):e18572. doi: 10.1111/jcmm.18572 (PMC11284121; doi:10.1111/jcmm.18572)
Supplement: Supplementary file 1 — Table S1. [file JCMM-28-e18572-s001.docx]

**Table S1.** Clinicopathological characteristics for the primary PCa cohort (n=48).

**Table S2.** Clinicopathological characteristics for the PCa metastases (n=22).
